# Decreased Metabolic Flexibility in Skeletal Muscle of Rat Fed with a High-Fat Diet Is Recovered by Individual CLA Isomer Supplementation via Converging Protective Mechanisms

**DOI:** 10.3390/cells9040823

**Published:** 2020-03-29

**Authors:** Giovanna Trinchese, Gina Cavaliere, Fabiano Cimmino, Angela Catapano, Gianfranca Carta, Claudio Pirozzi, Elisabetta Murru, Adriano Lama, Rosaria Meli, Paolo Bergamo, Sebastiano Banni, Maria Pina Mollica

**Affiliations:** 1 Department of Biology, University of Naples Federico II, 80126 Naples, Italy; giovanna.trinchese@unina.it (G.T.); gina.cavaliere@unina.it (G.C.); fabiano.cimmino@unina.it (F.C.); angelacatapano@me.com (A.C.); 2 Department of Pharmacy, University of Naples Federico II, 80131 Naples, Italy; claudio.pirozzi@unina.it (C.P.); adriano.lama@unina.it (A.L.); meli@unina.it (R.M.); 3 Department of Biomedical Sciences, University of Cagliari, Monserrato, CA 09042, Italy; giancarta@unica.it (G.C.); m.elisabetta.murru@gmail.com (E.M.); banni@unica.it (S.B.); 4 Institute of Food Sciences, National Research Council, 83100 Avellino, Italy; paolo.bergamo@isa.cnr.it

**Keywords:** CLA, metabolic flexibility, mitochondrial function

## Abstract

Energy balance, mitochondrial dysfunction, obesity, and insulin resistance are disrupted by metabolic inflexibility while therapeutic interventions are associated with improved glucose/lipid metabolism in skeletal muscle. Conjugated linoleic acid mixture (CLA) exhibited anti-obesity and anti-diabetic effects; however, the modulatory ability of its isomers (cis_9_, trans_11_, C9; trans_10_, cis_12_, C10) on the metabolic flexibility in skeletal muscle remains to be demonstrated. Metabolic inflexibility was induced in rat by four weeks of feeding with a high-fat diet (HFD). At the end of this period, the beneficial effects of C9 or C10 on body lipid content, energy expenditure, pro-inflammatory cytokines, glucose metabolism, and mitochondrial efficiency were examined. Moreover, oxidative stress markers, fatty acids, palmitoyletanolamide (PEA), and oleyletanolamide (OEA) contents along with peroxisome proliferator-activated receptors-alpha (PPARα), AKT, and adenosine monophosphate-activated protein kinase (AMPK) expression were evaluated in skeletal muscle to investigate the underlying biochemical mechanisms. The presented results indicate that C9 intake reduced mitochondrial efficiency and oxidative stress and increased PEA and OEA levels more efficiently than C10 while the anti-inflammatory activity of C10, and its regulatory efficacy on glucose homeostasis are associated with modulation of the PPARα/AMPK/pAKT signaling pathway. Our results support the idea that the dissimilar efficacy of C9 and C10 against the HFD-induced metabolic inflexibility may be consequential to their ability to activate different molecular pathways.

## 1. Introduction

Metabolic flexibility is the capacity for the organism to adapt fuel oxidation to nutrient availability and allows different tissues (liver, skeletal muscle, heart) to adjust their metabolism, managing nutrient sensing, uptake, transport, storage, and expenditure [1]. Mitochondria represent the functional core of metabolic flexibility [2]. Indeed, for the adaptation of tissue functions to nutrient availability, the modulation of oxidative phosphorylation activity is essential, according to substrate preference [3,4]. Deteriorated insulin-mediated substrate switching is the best example of metabolic inflexibility and it has a central role in obesity and in the development of insulin resistance [5]. Leptin and adiponectin are two adipokines that exert potent insulin-sensitizing effects, leading to a decrease in triglyceride content in the peripheral tissues (liver and skeletal muscle) and increase of fatty acid (FA) oxidation. Adiponectin acts via the activation of peroxisome proliferator-activated receptors-alpha (PPAR-α) while leptin through direct activation of adenosine monophosphate-activated protein kinase (AMPK) [6]. Interestingly, isolated skeletal muscle mitochondria from rats fed high-sugar or high-fat diets showed reduced metabolic flexibility, indicating that substrate preference is independent of cytosolic–mitochondrial communication and in fact is a consequence of inherent mitochondrial biochemical network interactions [7]. Intriguingly, several food components, along with diet and exercise, have been indicated as a promising approach to manage the main clinical complications of metabolic syndrome [8], but their efficacy in modulating metabolic flexibility has been only partially investigated. In particular, it is generally believed that dietary fat with an imbalanced ratio of saturated fatty acids (SFA)/polyunsaturated fatty acids (PUFA), the high n-6/n-3 polyunsaturated fatty acids (PUFA) ratio is associated with increasing obesity and metabolic disorders [9,10], while balancing the diet with monounsaturated fatty acids (MUFA) or n-3 PUFA may counteract inflammation [11] and insulin resistance [8]. Beside these PUFAs, several beneficial effects on lipid/glucose metabolism were also elicited by the intake of synthetic conjugated linoleic acid (CLA) composed by an equimolar mixture (1:1) of cis_9_,trans_11_ (C9) and trans_10_,cis_12_ (C10). These are the most studied CLA isomers and they may elicit distinct biological activities [12], acting through multiple pathways [13] and inducing beneficial effects via synergistic [14,15] or even independent activities [16]. The main dietary sources of the C9 isomer (C18:2, n-7) are meat and dairy products of ruminants, and this isomer is the most abundant and represents up to 80% of the total CLA in food [17]. Instead, while the C10 isomer (C18:2, n-6) represents 10% of the CLA found in ruminant meats and dairy products, it is mainly present in synthetic supplements and exhibits characteristic biological activities. When administered as an equimolar mixture, CLA reduces body fat accumulation and increases energy expenditure in several animal models, as well as in humans [18,19,20,21,22,23,24,25]. Previously, we demonstrated that C9 administration ameliorates ectopic lipid accumulation in the liver more efficiently than C10 and exerts a protective effect against diet-induced pro-oxidant and pro-inflammatory signs, likely via activation of the nuclear erythroid-derived 2-like-2 factor (Nrf2) and an improvement of hepatic mitochondrial function/efficiency [26]. Since skeletal muscle is the major targets of insulin action [27] and the key determinant of the resting metabolic rate [28], thus modulation of the mitochondrial function/efficiency in this tissue has been suggested as a potential approach to treat obesity and insulin resistance [29,30]. CLA was also demonstrated as an effective activator of both the AMPK [31], the key intracellular sensor of the energy state [32], and the PPARα [33], lipid sensors that modulate metabolic pathways in response to substrate disposal [34]. On the basis of the recognized involvement of AMPK in glucose and lipid metabolism [35], and PPARs’ ability to activate the expression of a range of metabolic and anti-inflammatory genes [34], we decided to evaluate the protective ability of individual CLA isomers on energy balance and body composition and on glucose/lipid metabolism and the pro-inflammatory effect associated with reduced mitochondrial metabolic flexibility in skeletal muscle in a rodent model of diet-induced obesity. Moreover, the different abilities of CLA isomers in modulating fatty acid and palmitoyletanolamide (PEA) and oleyletanolamide (OEA) content along with PPARα, AKT, and AMPK protein were also investigated.

## 2. Materials and Methods

### 2.1. Reagents

All chemicals were purchased by Sigma–Aldrich (St. Louis, MO, USA), unless otherwise specified. Pure C9 (cis_9_, trans_10,_ #1245) and C10 (trans_10_,cis_12_ #1249) were purchased from Matreya LLC (Pleasant Gap, State College, PA, USA).

### 2.2. Animals and Diets

Young male Wistar rats (60 days old; 350 ± 6 g; Charles River, Calco, Lecco, Italy) were individually caged in a temperature-controlled room and exposed to a daily 12 h–12 h light–dark cycle with free access to diet and drinking water. Rats were divided in two experimental groups according to a different dietary regimen: The first group was fed with a standard rodent diet (CD, n = 7) (15.88 kJ gross energy/g: 60.4% carbohydrates, 29% protein, 10.6% fat; Mucedola, Milan, Italy); the second was fed with a high-fat diet (HFD, n = 21) (20 kJ/g) in which 40% of the metabolizable energy was obtained from lard, and the remaining calories were starch (31%) and protein (29%). The HFD group was further divided in three subgroups (n = 7 each): One subgroup was fed with only HFD; the other two subgroups of HFD rats were daily administered with 30 mg of C9 or C10 isomer corresponding to approximately 80 mg/kg body weight or to 0.78 g/day, when expressed as a “human equivalent dose [36]. This dose was chosen because it is comparable with the reported daily intake in humans [37] and comprises the contribution of trans9-C18:1, which is converted in C9 by liver desaturases [38]. An additional group (n = 6) was sacrificed at the beginning of the study to establish baseline measurements of body composition.

After 4 weeks of treatment, before their sacrifice, the rats were anaesthetized by an intra-peritoneal injection of chloral hydrate (40 mg/100 g body weight) and blood was taken via the inferior cava vein and collected in heparin or EDTA-containing tubes. Hind leg muscles were removed, freed of excess fat and connective tissue, and were frozen and stored at −80 °C for further determination, if not immediately used.

### 2.3. Ethics Statement

Procedures involving animals and their care were conducted in conformity with international and national law and policies (EU Directive 2010/63/EU for animal experiments, ARRIVE guidelines, and the Basel declaration including the 3R concept). The procedures reported here were approved by the Institutional Committee on the Ethics of Animal Experiments of the University of Naples “Federico II”, and the Italian Ministry of Health (Permission n. 176/2005A)

### 2.4. Body Composition and Energy Balance

During the experimental period, body weight and food intake were monitored daily to calculate weight gain and gross energy intake. Spilled food and feces were collected daily for precise food intake calculation. Energy balance assessments were conducted over the 4 weeks of feeding by comparative carcass evaluation [39]. The gross energy density for the standard diet or high-fat diet (15.8 or 20.0 kJ/g, respectively), as well as the energy density of the feces and the carcasses, were determined by bomb calorimeter (Parr adiabatic calorimeter, Parr Instrument Co., Moline, IL, USA). The energy, fat, and protein contents in the animal carcasses were measured, according to Cavaliere et al. [40]. Metabolizable energy (ME) intake was determined by subtracting the energy measured in the feces and urine from the gross energy intake, which was determined from the daily food consumption and gross energy density. Gross energy efficiency was calculated as the percentage of body energy retained per ME intake, and energy expenditure was determined as the difference between ME intake and energy gain.

### 2.5. Measurement of Oxygen Consumption (VO_2_), Carbon Dioxide Production (VCO_2_), and Respiratory Quotient (RQ)

Upon an adaption period to the experimental environment, VO_2_ and VCO_2_ were recorded by a monitoring system (Panlab s.r.l., Cornella, Barcelona, Spain) composed of a four-chambered indirect open-circuit calorimeter, designed for continuous and simultaneous monitoring. VO_2_ and VCO_2_ were measured every 15 min (for 3 min) in each chamber for a total of 6 h (from 8:00 am to 14:00 pm). The mean VO_2_, VCO_2_, and RQ values were calculated by the “Metabolism H” software (Metaox, Metabolism V2.1).

Metabolism studies Plarform [41].

### 2.6. Evaluation of Biochemical Parameters in Blood

Blood samples were centrifuged at 1400 × *g* for 8 min at 4 °C. Plasma was removed and stored at 20 °C. Plasma insulin concentrations were measured with the use of enzyme-linked immunosorbent assay kits in a single assay to remove inter-assay variations (Mercodia AB, Uppsala, Sweden). Glucose levels were determined by a glucometer (Contour XT, Ascensia Diabetes Care, Milan, Italy). Basal fasting values of serum glucose and insulin were used to calculate the homoeostatic model assessment (HOMA) index as glucose (mg/dL) × insulin (mU/L)/405 [42]. Plasma concentrations of triglycerides and cholesterol, and non-esterified fatty acids (NEFAs) were measured by the colorimetric enzymatic method using commercial kits (SGM Italia, Rome, Italy and Randox Laboratories ltd., Crumlin, United Kingdom). Commercially available ELISA kits were used to determine serum adiponectin and leptin (B-Bridge International, Mountain View, CA, USA), interleukin-1α (IL-1α), interleukin-10 (IL-10), and tumor necrosis factor-α (TNF-α) (Thermo Scientific, Rockford, IL, USA; Biovendor R and D, Brno, Czech Republic).

### 2.7. Mitochondria Preparation and Analysis

Limb leg muscle aliquots were freed of excess fat and connective tissue, finely minced, and washed in a medium containing 100 mM KCl, 50 mM Tris-HCl, pH 7.5, 5 mM MgCl_2_, 1 mM EDTA, 5 mM EGTA, 0.1% (w/v) fatty acid free bovine serum albumin (BSA). Tissue fragments were homogenized with the above medium (1: 8, w/v) in a Potter Elvehjem homogenizer (Heidolph, Kelheim, Germany) set at 500 rpm (4 strokes = min) and filtered through sterile gauze and finally centrifuged (3000 × *g*, 10 min, 4 °C). The resulting supernatant was discarded, and the pellet was resuspended and centrifuged at 500 × *g* for 10 min. The supernatant was centrifuged (3000 × *g*, 10 min, 4 °C) and the pellet, containing the mitochondrial fraction, was washed once and resuspended in suspension medium [43]. Mitochondrial oxygen consumption was evaluated as previously reported [44]. Oxygen consumption was polarographically measured by a Clark-type electrode (Yellow Springs Instruments, Yellow Springs, OH, USA) at 30 °C. In detail, isolated mitochondria (0.5 mg protein/mL) were incubated in a medium containing 30 mM KCl, 6 mM MgCl_2_, 75 mM sucrose, 1 mM EDTA, 20 mM KH_2_PO_4_ pH 7.0, and 0.1% (w/v) fatty acid-free BSA. In the presence of 10 mM succinate, 3.75 µM rotenone, and 0.6 mM ADP, state 3 oxygen consumption was measured. State 4 was obtained in the absence of ADP. The rate of mitochondrial fatty acid oxidation was assessed in the presence of malate (2.5 mM), palmitoyl-L-carnitine (40 µM), and ADP (0.6 mM). The respiratory control ratio (RCR) was calculated as the ratio between states 3 and 4. In control experiments, we assured the quality of our mitochondrial preparation by checking that contamination of mitochondria by other ATPase-containing membranes was lower than 10%, and the addition of cytochrome c (3 nmol/mg protein) only enhanced the state 3 respiratory rate by approximately 10%. The degree of coupling was determined in mitochondria as previously reported [45] by applying Equation (11) by Cairns et al. [46]: Degree of coupling = $\sqrt{1}-\frac{\left(\mathrm{Jo}\right)\mathrm{sh}}{\left(\mathrm{Jo}\right)\mathrm{unc}}$, where (Jo)_sh_ represents the oxygen consumption rate in the presence of oligomycin that inhibits ATP synthase, and (Jo)_unc_ is the uncoupled rate of oxygen consumption induced by carbonyl cyanide 4-(trifluoromethoxy)phenylhydrazone (FCCP), which dissipates the trans-mitochondrial proton gradient. (Jo)_sh_ and (Jo)_unc_ were measured as above using succinate (10 mmol/L) + rotenone (3.75 μmol/L) in the presence of oligomycin (2 μg/mL) or FCCP (1 μmol/L), respectively, both in the absence and in the presence of palmitate at a concentration of 50 μmol/L. Carnitine-palmitoyl-transferase (CPT) activity was followed spectrophotometrically as CoA-sH production by the use of 5,5′-dithiobis (nitrobenzoic acid) (DTNB) and as substrate palmitoyl-Coa 10 μM. The medium consisted of 50 mM KCl, 10 mM Hepes (pH 7.4), 0.025% Triton X-100, 0.3 mM DTNB, and 10–100 pg of mitochondrial protein in a final volume of 1.0 mL. The reaction was followed at 412 nm at 25 °C in a thermostated spectrophotometer and enzyme activity was calculated from an E412 = 13,600/(M × cm) [47]. The rate of mitochondrial H_2_O_2_ release was assayed by following the linear increase in fluorescence (excitation 312 nm and emission 420 nm) due to the oxidation of homovanillic acid in the presence of horseradish peroxidase [48]. Superoxide dismutase (SOD) specific activity was measured in a medium containing 0.1 mM EDTA, 2 mM KCN, 50 m KH_2_PO_4_, pH 7.8, 20 mM cytochrome c, 5 m xanthine, and 0.01 U of xanthine oxidase. Enzyme activity was measured spectrophotometrically (550 nm) at 25 °C, by monitoring the decrease in the reduction rate of cytochrome c by superoxide radicals, generated by the xanthine–xanthine oxidase system. One unit of SOD activity is defined as the concentration of enzyme that inhibits cytochrome c reduction by 50% in the presence of xanthine and xanthine oxidase [49]. Aconitase activity in skeletal muscle was carried out in a medium containing 30 mM sodium citrate, 0.6 mM MnCl_2_, 0.2 mM NADP, 50 mM TRIS-HCl pH 7.4, and 2 units of isocitrate dehydrogenase. The formation of NADPH was followed spectrophotometrically (340 nm) at 25 °C. The level of aconitase activity measured equals active aconitase (basal level). Aconitase inhibited by reactive oxygen species (ROS) in vivo was reactivated so that the total activity could be measured by incubating mitochondrial extracts in a medium containing 50 mM dithiothreitol, 0.2 mM Na_2_S, and 0.2 mM ferrous ammonium sulphate [50].

### 2.8. Lipid Extraction and Analysis

The total lipid content in limb muscle was estimated by using the Folch method [51]. Briefly, muscle was weighed, chopped into small pieces, thoroughly mixed, and upon the addition of 2 parts of water (w/w), it was homogenized by a Polytron homogenizer. Homogenate aliquots were weighted, and the lipid content was determined gravimetrically after extraction in chloroform–methanol and evaporation by a rotating evaporator and their amount was finally expressed as mg/g of tissue. Lipid peroxidation extent was assessed using the thiobarbituric acid assay and its amount was expressed as pmole of TBARS/mg of protein [52].

### 2.9. Analysis of Fatty Acids, Endocannabinoids, and Congeners

Fatty acid analysis was conducted from the total lipids previously extracted from tissues by the method of Folch [51]. Aliquots of chloroform were dried and mildly saponified as previously described [53] in order to obtain free fatty acids for HPLC analysis. The separation of unsaturated fatty acids was carried out with an Agilent 1100 HPLC system (Palo Alto, CA, USA) equipped with a diode array detector, as previously reported [54], and their amount was finally expressed as % of total fatty acids. Endocannabinoid and congener quantification are described in [55]. Deuterated EC and congeners were added as internal standards to the samples before extraction. Analyses were carried out by liquid chromatography, atmospheric pressure chemical ionization, and MS (LC–APCI–MS) (Palo Alto, CA, USA), using selected ion monitoring (SIM) at M + 1 values for the compounds and their deuterated homologs and their amount was finally expressed as % of total fatty acids. The n-3 HUFA (Highly Unsaturated Fatty Acid) score was calculated as the percentage of the sum of n-3 FAs with 20 or more carbon atoms, and three or more double bonds, namely eicosapentaenoic acid (C20:5 n-3 EPA), docosahexaenoic acid (C22:6n-3, DHA), and docosapentaenoic acid (22:5 n-3, DPAn-3) divided by the sum of total FAs with 20 or more carbon atoms and more than three double bonds namely. EPA + DHA + DPAn-3 + diomogamma linoleic (C20:3, DGLA) + arachidonic acid (C20:4 n6, AA) + adrenic acid (22:4n-6, AdA) + osbond acid (C22:5n6 o DPAn-6) + eicosatrienoic acid (20:3n-9, ETA):

n-3 HUFA score = (EPA + DHA + DPAn-3)/(EPA + DHA + DPAn-3 + DGLA + AA + AdA + DPAn-6 + ETA) × 100.

### 2.10. Western Blot Analysis

Skeletal muscle (100 mg wet tissue), obtained from the various experimental groups, was lysed on ice in lysis buffer (20 mM Tris–HCl, pH 7.5, 10 mM NaF, 150 mM NaCl, 1% Nonidet P-40, 1 mM phenylmethylsulphonyl fluoride, 1 mM Na_3_VO_4_, leupeptin, and trypsin inhibitor 10 µg/mL). After 40 min, total protein lysates were obtained by centrifugation at 14,000 × *g* for 15 min at 4 °C. Protein concentrations were estimated by the Bio-Rad protein assay using BSA as the standard and an equal amount of protein (50 µg) were subjected to SDS-PAGE and transferred onto a nitrocellulose membrane (Amersham Biosciences, Little Chalfont, Buckinghamshire, UK) using a Bio-Rad Transblot (Bio-Rad, Milan, Italy). Membranes were blocked at room temperature in milk buffer (1X PBS, 10% w/v nonfat dry milk, 0.1% v/v Tween-20) and then incubated at 4 °C overnight with anti-PPARα (diluition 1:500), anti-phosphoAKT (dilution 1:1000) and anti-AKT (dilution 1:1000), anti-phospho-AMPKα (dilution 1:500) and AMPK (dilution 1:1000) (Cell Signaling, Danvers, MA, USA), and anti-glucose transporter 4 (GLUT4) (Novus Biologicals, Centennial, CO, USA). Subsequently, the membranes were incubated for 60 min at room temperature with peroxidase-conjugated appropriate antibodies (Jackson ImmunoResearch Laboratories, Baltimore Pike, West Grove, PA, USA). Western blot for glyceraldehyde 3-phosphate dehydrogenase (GAPDH, dilution 1:5000) (Sigma-Aldrich, Milan, Italy) was performed to ensure equal sample loading.

### 2.11. Statistical Analyses

All data were presented as means ± SEM. Differences among groups were compared by ANOVA followed by the Bonferroni post hoc test to correct for multiple comparisons. Differences were considered statistically significant at *p* < 0.05. All analyses were performed using GraphPad Prism 5.0 (GraphPad Software, San Diego, CA, USA).

## 3. Results

### 3.1. C9 Supplementation Decreases Body Lipid Accumulation and Increases Energy Expenditure More Efficiently than C10

**Figure 1:**
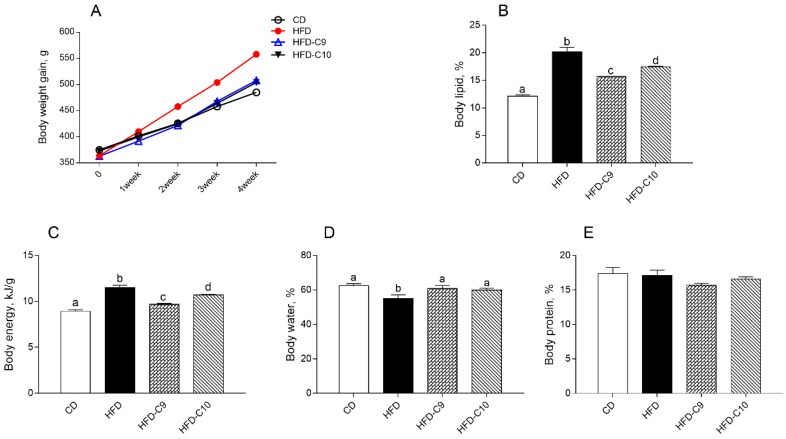
cis_9_, trans_11_ (C9) or trans_10_, cis_12_ (C10) isomer intake influences body weight gain and composition in high fat diet (HFD)-fed rats. Body weight gain during the study period (4 weeks) (**A**), body lipid (**B**), energy (**C**), water (**D**), and protein (**E**) were reported. Data were expressed as the means ± SEM n = 7 animals/group. Differing superscripted letters indicate statistically significant differences (*p* < 0.05).

The body weight gain, body lipid, and body energy of animals fed the HFD were significantly higher than those of rats fed the standard diet (Figure 1A–C). The administration of CLA significantly reduced these parameters (C9 > C10) as compared to the HFD group, and a significant higher body water content was accordingly exhibited by HFD-C9 and HFD-C10 rats in comparison with HFD animals (Figure 1D). No significant difference was observed in body protein levels between the different groups (Figure 1E).

**Figure 2:**
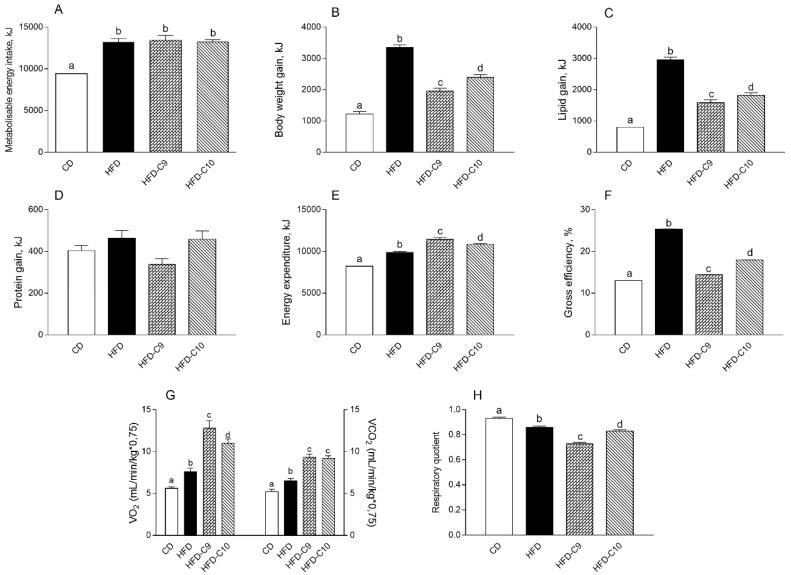
cis_9_, trans_11_ (C9) supplementation modulates energy expenditure and efficiency in high fat diet (HFD)-fed rats more efficiently than trans_10_, cis_12_ (C10). Metabolizable energy intake (**A**), body weight, lipid and protein gain (**B**–**D**), energy expenditure (**E**) and efficiency (**F**), VO_2_ = oxygen consumption and VCO_2_ = carbon dioxide production (**G**), and the respiratory quotient (calculated as the ratio VCO_2_/VO_2_) (**H**) were measured in different animal groups. Data were expressed as the means ± SEM n = 7 animals/group. Differing superscripted letters indicate statistically significant differences (*p* < 0.05).

HFD treatment resulted in a significantly higher metabolisable energy intake, body weight gain, lipid gain, and gross efficiency, as compared to the control group (Figure 2A–C,F). Both CLA supplementations had undetectable effects on metabolizable energy (Figure 2A) while they showed a significant decrease of body weight, lipid gain, and gross efficiency (C9 > C10) (Figure 2B,C,F) in comparison with the HFD group. No differences in body protein gain were observed between different groups (Figure 2D). In addition, HFD-C9 and HFD-C10 animals showed significantly higher energy expenditure and O_2_ consumption/CO_2_ production in comparison with control and HFD-fed animals (Figure 2E,G). Taken together, these effects indicate the more marked capability of C9 in decreasing body energy, body lipid accumulation, and gross efficiency effects, compared to C10. Data showing the lower respiratory quotient (RQ) in the C9-treated group, compared to the other treatment, are consistent with data showing lower lipid gain accumulation and an improved ability to utilize fat as metabolic fuel (Figure 2H).

### 3.2. Supplementation with C9 or C10 Differently Modulates Lipid and Glucose Metabolism and Inflammation

**Figure 3:**
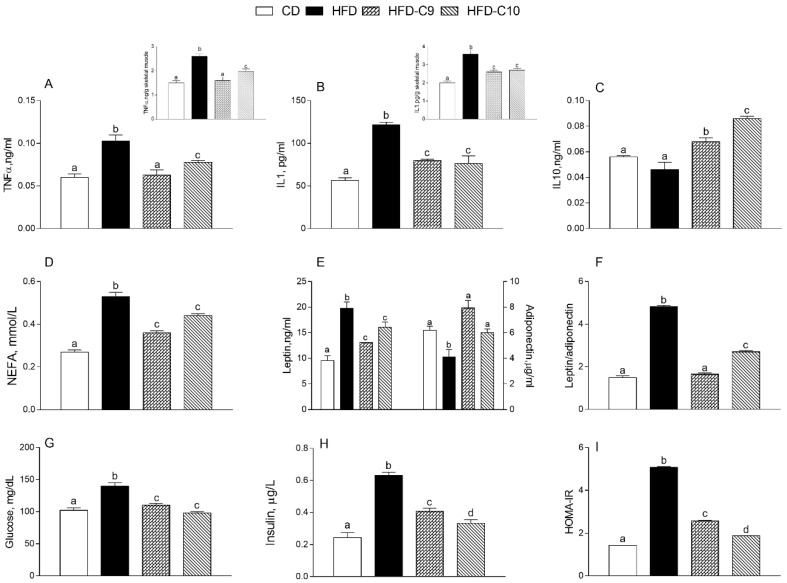
cis_9_, trans_11_ (C9) or trans_10_, cis_12_ (C10) isomer supplementation exhibits different efficacy in reducing proinflammatory markers and affected serum metabolic parameters of high fat diet (HFD)-fed rats. Proinflammatory cytokines, such as tumor necrosis factor (TNF-α) (**A**) and interleukin-1 (IL-1) (**B**), were measured in serum and in muscle (upper panels). Anti-inflammatory markers IL-10 were also determined in serum (**C**). Metabolic parameters, such as non-esterified fatty acids (NEFA) (**D**), leptin, adiponectin, and their ratio (**E**–**F**), glucose (**G**), insulin (**H**), and the HOMA index (**I**) are shown. Data were expressed as the means ± SEM n = 7 animals/group. Differing superscripted letters indicate statistically significant differences (*p* < 0.05).

As expected, all the considered hyperlipidemia and proinflammatory markers were significantly increased in the blood, and in skeletal muscle, of HFD-treated rats in comparison with controls (Figure 3). Interestingly, both CLA supplementations limited the detrimental effects of HFD. Although they reduced the level of proinflammatory cytokines in blood (IL-1 and TNF-α) and increased anti-inflammatory cytokine IL-10 (Figure 3A–C), similar effects were shown in skeletal muscle (Figure 3A,B upper panels). In particular, HFD-C9 rats exhibited a more remarkable reduction in lipid metabolism and in pro-inflammatory cytokines as compared to HFD-C10 and HFD-fed rats.

Notably, both isomers of CLA significantly reduced NEFA and leptin concentrations (Figure 3D,E) as compared to HFD-fed animals. In addition, the adiponectin concentration in HFD-C9- and HFD-C10-treated animals was comparable to that measured in controls (Figure 3D,E) and, consequently, the HFD-induced alteration of the leptin/adiponectin ratio was reduced by C10 or restored by C9 supplementation (Figure 3F). 

As expected, HFD intake increased glycaemia and insulin levels as compared to controls, which was significantly reduced by both CLA supplementations (Figure 3G,H). Similarly, HFD-C9 and HFD-C10 groups exhibited a marked reduction of the HOMA index as compared to HFD-fed animals, and C10 supplementation better decreased the HOMA index (Figure 3I).

### 3.3. C9 Supplementation Increases Mitochondrial Fatty Acid Oxidation and Reduces Oxidative Stress More Efficiently than that with C10

**Figure 4:**
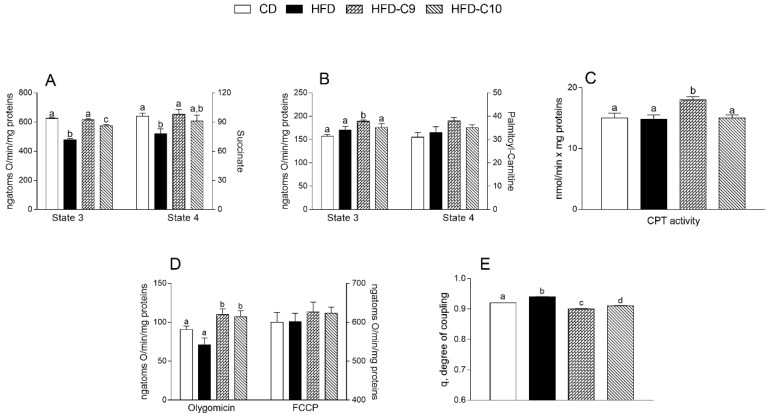
cis_9_, trans_11_ (C9) increases mitochondrial fatty acid oxidation and affects the coupling efficiency in the skeletal muscle mitochondria of high fat diet (HFD)-fed rats more efficiently than trans_10_, cis_12_ (C10). The skeletal muscle mitochondrial respiration rates were evaluated in the presence of succinate (**A**) or palmitoyl-carnitine (**B**) as substrates. CPT activity was measured in isolated mitochondria (**C**). Oxygen consumption in the presence of oligomycin or uncoupled by carbonyl cyanide 4-(trifluoromethoxy)phenylhydrazone (FCCP) (**D**) and the degree of coupling (**E**) were also shown. Data were expressed as the means ± SEM n = 7 animals/group. Differing superscripted letters indicate statistically significant differences (*p* < 0.05)**.**

The skeletal muscle mitochondrial respiration rates were measured in the presence of succinate or palmitoyl-carnitine as substrates. In the presence of succinate, state 3 and state 4 mitochondrial oxygen consumptions were significantly reduced in the HFD group when compared to the control group. An increase of the respiration rate was observed in HFD-C9 and HFD-C10 rats compared to the animals that received HFD alone (HFD-C9 > HFD-C10) (Figure 4A). In the presence of palmitoyl-carnitine, the state 3 respiration rates were similar in HFD-fed groups compared to control animals. Only the C9 supplementation determined a significantly higher oxygen consumption (Figure 4B), paralleled by higher CPT activity (Figure 4C), than the control group. No significant differences were observed in state 4 respiration rates among all groups (Figure 4B). The respiratory control ratio (RCR) values were indicative of high-quality mitochondrial preparations (data not shown).

Oligomycin state 4 respiration was significantly higher in CLA-supplemented animals compared to HFD or control rats while no variation was found in maximal FCCP-stimulated respiration between the groups (Figure 4D). As a consequence, skeletal muscle mitochondrial energetic efficiency, assessed as the degree of coupling, was lower in CLA-supplemented animals than the HFD group, with a more significant reduction in C9-supplemented animals than the C10-supplemented group (Figure 4E).

**Figure 5:**
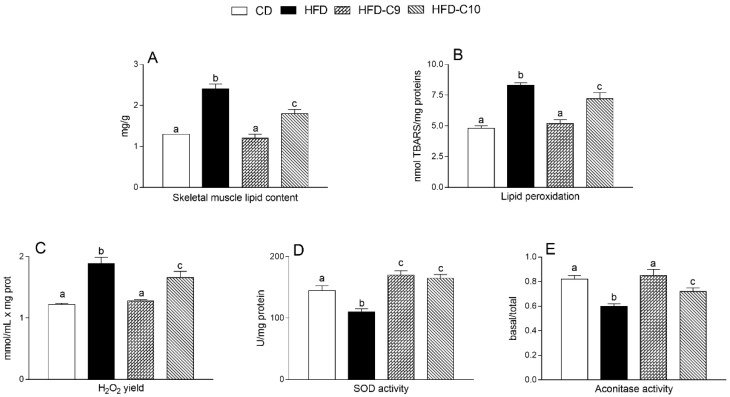
cis_9_, trans_11_ (C9) or trans_10_, cis_12_ (C10) isomer supplementation exhibits different efficacy in reducing lipid content and oxidative stress marker alteration in the skeletal muscle of high fat diet (HFD)-fed rats. The lipid amount (**A**) and lipid peroxidation index (**B**) were determined in the skeletal muscle of all rat groups. Mitochondrial H_2_O_2_ yield (**C**), superoxide dismutase (SOD) (**D**), and basal/total aconitase activity (**E**) were reported. Data were expressed as the means ± SEM n = 7 animals/group. Differing superscripted letters indicate statistically significant differences (*p* < 0.05).

Lipid content and oxidative stress markers were also investigated in the muscle tissue of differently treated rats. As expected, HFD intake was associated with a significant rise of the lipid amount, lipid peroxidation index (thiobarbituric acid reactive substances, TBARS), and H_2_O_2_ yield (an indirect index of mitochondrial superoxide production) as compared to controls and their level was more markedly decreased by C9 rather that C10 supplementation (Figure 5A–C). HFD intake was also associated with a significant decline of superoxide dismutase-2 (SOD2) and basal/total aconitase activity (sensitive marker of oxidative stress) that was efficiently reduced by both C9 and C10 supplementation or recovered by C9 but not by C10 treatment, respectively (Figure 5D,E).

### 3.4. C10 Supplement Improves Glucose Homeostasis by Modulating PPAR-α/AMPK/AKT and GLUT4 Pathway

**Figure 6:**
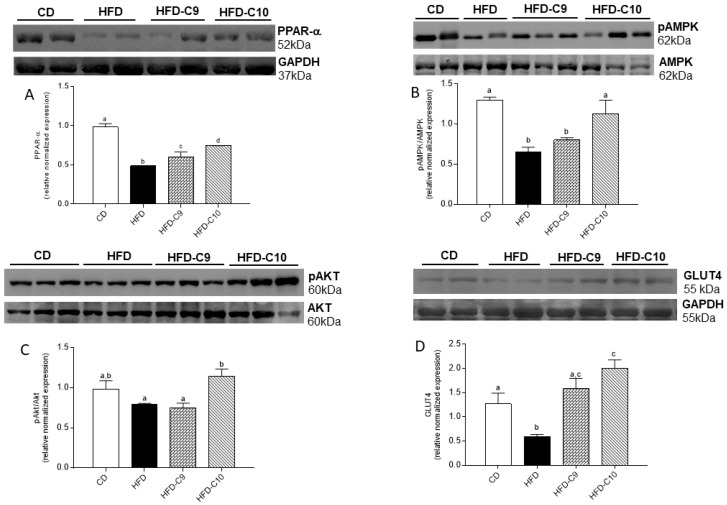
cis_9_, trans_11_ (C9) or trans_10_, cis_12_ (C10) isomer supplementation exhibits different efficacy in improving high fat diet (HFD)-induced alteration of peroxisome proliferator-activated receptors-alpha (PPAR-α)/adenosine monophosphate-activated protein kinase (AMPK)/AKT and glucose transporter (GLUT4) expression. Skeletal muscle PPAR-α expression (**A**), phosphorylated (p) AMPK-to-AMPK (**B**) and pAKT-to-AKT (**C**) ratios, and GLUT4 expression (**D**) were evaluated by Western blot and densitometric analysis. Data were expressed as the means ± SEM n = 4 animals/group. Differing superscripted letters indicate statistically significant differences (*p* < 0.05).

To determine the mechanism underlying the modulatory effect of individual CLA isomers, on the HFD-induced alteration of glucose and lipid metabolism, PPARα, AKT, AMPK, and GLUT4 protein expression were evaluated in the skeletal muscle of all animal groups (Figure 6). The results clearly indicated that C10 was able to markedly restore the HFD-induced reduction of PPARα expression and the phosphorylation of AKT and AMPK, as compared to HFD-C9 animals (Figure 6A–C). Moreover, the increased expression of GLUT4, the main glucose transporter in muscle, in both CLA-treated groups was indicative of their ability in improving glucose metabolism in HFD rats (Figure 6D).

### 3.5. C9 and C10 Supplementation Increases CLA and n-3 PUFA Parameters in Liver and Muscle

**Figure 7:**
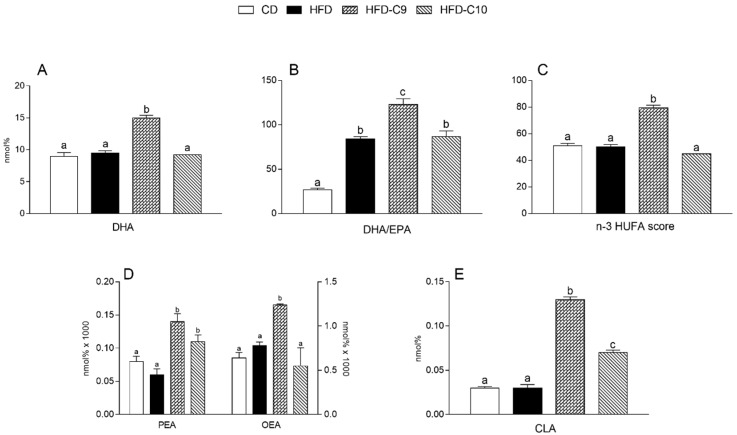
cis_9_, trans_11_ (C9) or trans_10_, cis_12_ (C10) isomer supplementation exhibits different efficacy in modulating the alteration of fatty acid composition in skeletal muscle associated with high fat diet (HFD) intake. Tissue levels of docosahexaenoic acid (DHA) (**A**), DHA/ EPA (eicosapentaenoic acid) ratio (**B**), n-3 highly unsaturated fatty acid (HUFA) score (**C**), palmitoyletanolamide (PEA) and oleyletanolamide (OEA) (**D**) and conjugated linoleic acid (CLA) (**E**) amounts were measured in the skeletal muscle of differently treated animals. Data were expressed as the means ± SEM n = 4 animals/group. Differing superscripted letters indicate statistically significant differences (*p* < 0.05).

Skeletal muscle DHA and the n-3HUFA score were not altered by HFD intake and, interestingly, the C9 treatment was associated with their significant increase compared to other groups (Figure 7A,C). The DHA/EPA ratio in the skeletal muscle of HFD and HFD-C10 group rats was significantly higher in comparison with controls and it was more markedly increased by C9 supplementation (Figure 7B). We also observed a significant increase of PEA in both CLA-treated groups, whereas the OEA level was significantly higher only in HFD-C9 animals (Figure 7D). As expected, C9 and C10 supplementation was accompanied by CLA accumulation in skeletal muscle (C9 > C10) compared to HFD or untreated animals (Figure 7E).

**Figure 8:**
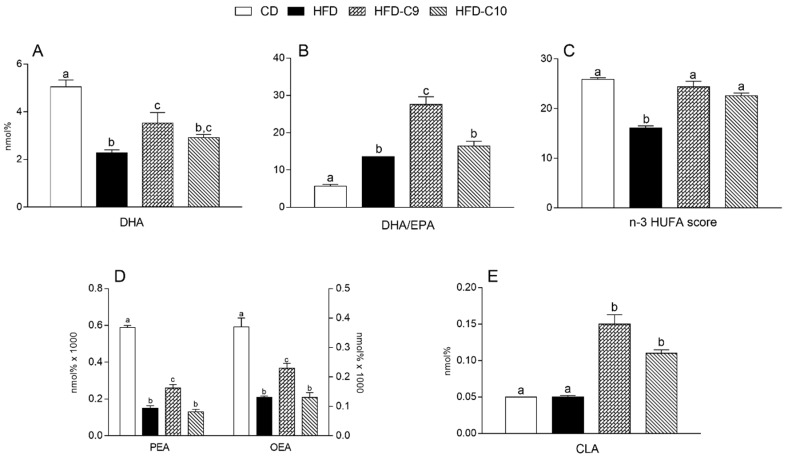
cis_9_, trans_11_ (C9) or trans_10_, cis_12_ (C10) isomer supplementation exhibits different efficacy in modulating the alteration of fatty acid composition of liver skeletal muscle associated with high fat diet (HFD) intake. Tissue levels of DHA (**A**), DHA/EPA ratio (**B**), n-3 HUFA score (**C**), PEA and OEA (**D**), and CLA (**E**) amounts were measured in the liver of differently treated animals. Data were expressed as the means ± SEM n = 4 animals/group. Differing superscripted letters indicate statistically significant differences (*p* < 0.05).

As shown in the Figure 8, HFD treatment significantly reduced DHA and the n-3 HUFA score in the liver, and their levels were improved in the HFD-C9 and HFD-C10 groups (Figure 8A,C). Similar to the skeletal muscle, the DHA/EPA ratio in the liver of HFD and HFD-C10 rats was significantly higher in comparison with controls and it was more markedly increased by C9 supplementation (Figure 8B). OEA and PEA concentrations were significantly reduced in the liver of all the groups fed with HFD compared to controls, but C9 supplementation led to a significant increase of their level in comparison with HFD animals (Figure 8D). Similar to the muscle, C9 and C10 supplementation was accompanied by CLA accumulation (C9 > C10) compared to HFD or untreated animals (Figure 8E).

## 4. Discussion

The presented results confirm the different biological (anti-inflammatory and antioxidant) effects elicited by individual CLA isomers in this animal model of diet-induced obesity [26] and extend their different modulatory effects on metabolic flexibility in skeletal muscle. The recognized role of skeletal muscle in metabolic flexibility, due to the association of muscular mitochondrial dysfunction with insulin resistance [56], prompted us to evaluate individual CLA isomers’ (C9 and C10) efficacy in modulating mitochondrial function and efficiency in this tissue. The current results show, for the first time, that CLA isomer supplementation exhibits beneficial effects on several typical features of HFD-induced metabolic inflexibility (i.e., increased metabolic efficiency, weight gain, and body lipid levels; glucose and lipid homeostasis disruption; pro-inflammatory effects) via different mechanisms and with distinct efficacy. 

Body lipid accumulation in white adipose tissue and ectopic triglyceride storage in the skeletal muscle of HFD-fed rats has been reported to be consequential to impaired metabolic flexibility and decreased energy expenditure resulting from insufficient mitochondrial lipid oxidation in these animals [44]. One of the underlying mechanisms of impaired fatty acid metabolism in skeletal muscle may lead to decreased expression of muscle CPT1, or more pronounced inhibition of its activity by malonyl-coenzyme A, causing decreased mitochondrial uptake and oxidation of FA [57]. Our data show that, despite the comparable metabolizable energy intake in all rats fed the HFD, the beneficial effects resulting from both CLA supplementation on body weight and lipid accumulation can be, at least in part, explained by the increased energy expenditure and decline of the energy efficiency and RQ index (C9 > C10). This last parameter, reflecting the carbohydrate to fatty acid oxidation ratio, indicates the higher use of fatty acids as a fuel source in these animals, in comparison with the other groups. These data are consistent with previous studies showing C9 induced an improvement of the lipid oxidation in the liver [26] or in adipose tissue in vivo [58]. Moreover, the antilipidemic effect associated with CLA isomer intake (C9 > C10) is supported by an increased mitochondrial respiratory capacity and decreased mitochondrial efficiency in the skeletal muscle compared to HFD rats. As expected, HFD feeding, alone, reduces mitochondrial respiratory capacity, as indicated by the decrease in the succinate-state 3 oxygen consumption rate and increased oxidative stress [44]. Despite regular mitochondrial fatty acid oxidation capacity being maintained, as a result of a diet-induced increase of the free fatty acid (FFA) uptake, mitochondrial lipid oxidation is likely not sufficient to handle the greater FFAs load, resulting in metabolic inflexibility with a consequent increase in the body lipid depot in white adipose tissue and in ectopic triglycerides’ storage in the skeletal muscle of HFD-fed rats. Such lipid accumulation was decreased in CLA-treated animals (C9 > C10). In fact, in the HFD-C9 group, the enhancement of fatty acid oxidation was supported by increased CPT activity, which would further increase the entry of long-chain FFA into the mitochondria. In addition, the reduced mitochondrial efficiency in both CLA-treated groups (C9 > C10) likely results from a decreased degree of coupling. This decline implies that more substrates need to be burned to obtain the same amount of ATP, eliciting lipid oxidation rather than deposition as also indicated by lower NEFA serum levels and triglycerides accumulation in skeletal muscle.

Moreover, mitochondrial impairment triggered by HFD intake contributes to excessive ROS production (H_2_O_2_ production and inhibition of aconitase and SOD activity) and raised lipid peroxidation products (TBARS). The production of a large amount of ROS in the HFD group occurs via a concomitant increase in the β-oxidation rate (which enhances nicotinamide adenine dinucleotide (NADH) and flavine adenine dinucleotide (FADH_2_) generation and thus electron delivery to the respiratory chain) and respiratory chain impairment (as indicated by the decrease in succinate state 3 oxygen consumption, which could partially block the electron flow within the respiratory chain). Interestingly, although CLAs supplementation increases the mitochondrial oxidative capacity, nevertheless, ROS formation is minimized by the decline in mitochondrial coupling, which is known to maintain the membrane potential below the critical threshold for ROS production [59]. In this context, decreased oxidative stress in CLA-treated rats (C9 > C10) is likely a result of the modulation of mitochondrial proton leakage and improved antioxidant defenses. Altogether, these data suggest that CLA improves metabolic flexibility, challenged by HFD feeding, adjusting the energy metabolism at the muscular level by modulating the respiratory capacity, fatty acid oxidation, oxidative stress, and mitochondrial efficiency. Alteration of the mitochondrial function, linked to increased ROS production, triggers catabolic signaling pathways, leading to muscle atrophy and sarcopenia [60,61]. Muscle atrophy occurs in several pathological conditions, like diabetes, renal and hearth failure, cancer, and aging [62]. Thus, the attenuation of mitochondrial damage by CLA supplementation may represent a valuable therapeutic strategy to counteract sarcopenia.

CLA is a strong ligand of PPARα, a transcriptional factor that controls the expression of genes involved in fatty acid metabolism, including fatty acid transport, as well as catabolism (particularly mitochondrial fatty acid oxidation) or storage [63,64], and it is believed to exert some of its metabolic effects by activating this receptor [65]. A unifying mechanism of action of the beneficial effects reported here may be re-conducted to selective tissue PPARα activation by CLA isomers. Previously, we showed that C9 is able to increase PPARα expression in the liver [26]. Here, we demonstrated that PPARα protein increased in the muscle of rats fed C10. Consequently, it is feasible that the metabolic effect of C9 on fatty acid metabolism (i.e., increased n-3 HUFA score, DHA/EPA ratio, and DHA biosynthesis) may occur via the activation of PPARα in the liver and then exported in the muscle. Similar results were found in humans [66], where the intake of naturally enriched C9 cheese significantly increased plasma DHA and the n-3 HUFA score and PPARα gene expression, which is also involved in DHA biosynthesis [67]. Further experiments will be carried out to evaluate the expression of a subset of down-stream genes by activating PPAR-α and to investigate the modulatory role of PPAR-δ in lipid-induced alteration of mitochondrial β oxidation in skeletal muscle.

In addition, we recently showed that the dietary intake of CLA induced the biosynthesis of OEA and PEA in the liver of obese Zucker rats associated with reduced hepatic lipid deposition [68]. OEA and PEA are natural amides of oleic acid (OA, 18:1) and palmitic acid (PA, 16:0), respectively. It has been shown that OEA reduces food intake, body weight gain, and the content of triacylglycerol [69], and stimulates lipolysis and fatty acid oxidation in the liver and in the adipose tissue [70]. Intriguingly, we recently showed that PEA improves metabolic flexibility in mice fed a high-fat diet [71]. The presented data showed a major increase of PEA and OEA with C9, suggesting that CLA induced an increase of the n-3 HUFA score, along with PEA and OEA, through sustained PPARα activity [72]. In fact, PEA and OEA are known to play an important role in the modulation of many biological functions, including energy balance, inflammation, and insulin resistance [73,74], likely via the activation of PPARα. PPARα is expressed mostly in tissues with high rates of fatty acid oxidation and is involved in the regulation of insulin action while its modulatory role in glucose metabolism remains controversial. Indeed, it was demonstrated that PPARα-null mice are protected from insulin resistance induced by a high-fat diet [75]. The lack of PPARα in null mice is associated, in the fasted state, with increased whole-body glucose use and GLUT4 content in white adipose tissue but not in brown fat or muscles, suggesting that this regulation could be explained by the major role played by the PPARα absence in the brain rather than in the liver [76]. In another study, adipokines involvement was evidenced in insulin-mediated skeletal muscle glucose uptake and GLUT4 expression [77]. In accordance with this assumption, our results highlight that both CLA isomer supplementations are able to reduce inflammation and to counteract leptin and adiponectin alterations, adipokines that are involved in the control of energy homeostasis and inflammation [78] and in the regulation of glucose and lipid metabolism, through AMPK activation [79,80].

In a recent review, Pariza assumed that the CLA ability to activate so many biological mechanisms could be the result of the combined action of individual isomers [81]. In particular, t10,c12-CLA exhibit preeminent anti-obesity and anti-diabetic effects to modulate lipid metabolism and glucose tolerance while the c9,t11-CLA isomer shows preeminent anti-cancer [13] and anti-inflammatory activities [82,83]; therefore, the mechanisms activated by the CLA mixture may be the resultant of both individual and combined activity of the distinct isomers. 

Here, for the first time, we demonstrated that CLA isomers are able to significantly reduce glucose and insulin levels, restoring glucose homeostasis and improving the HOMA index (C10 > C9). In particular, on the basis of the recognized role of AMPK, as an energy sensor involved in the regulation of both lipid and carbohydrate homeostasis, the more marked efficacy of C10 treatment is likely correlated to its ability to activate the insulin-responsive pathway in the skeletal muscle, via AMPK, p-AKT, and GLUT4 expression, in accordance with previous data [78,79,84,85]. On the other hand, the protective effects of C9 on glucose metabolism are likely related to its ability to improve redox homeostasis through modulation of mitochondria ROS emissions and Nrf2 activation [86]. Studies aimed at evaluating Nrf2′s involvement in the biological effects elicited in skeletal muscle are in progress. 

## 5. Conclusions

CLA’s efficacy in modulating mitochondrial function, oxidative stress, and inflammatory state in HFD-treated animals may be interpreted as the result of converging protective mechanisms against diet-induced metabolic inflexibility in skeletal muscle. In particular, C9 intake ameliorates several pathological signs, mainly promoting inefficient metabolism (generating heat instead of ATP) and reducing ROS generation in mitochondria and increasing the n-3 HUFA score, PEA, and OEA via PPARα activation in the liver and next in the muscle. On the other hand, the more marked efficacy of C10 on the inflammatory state and glucose homeostasis in the muscle is likely consequential to its modulatory effect on the PPARα/AMPK/pAkt signaling pathway.

## 6. Strength and Limitations

Most of the animal studies aiming to investigate the CLA metabolic effects used mixtures (1:1) of C9 and C10 isomers. This is the first study analyzing the effects of the two isomers on metabolic flexibility in skeletal muscle. We demonstrated that the beneficial effects exhibited by CLA isomers can be attributed to the activation of different regulatory pathways. Further experiments will be needed to investigate the different effects of dietary C9 or C10 supplementation in modulating cellular and mitochondrial ROS production [87,88,89] and to prevent oxidative DNA damage [90]. Moreover, additional experiments will be necessary for better investigating the involvement of additional genes metabolically linked to PPAR-α (i.e. CPT and UCP3) and to the observed changes in fatty acid composition. In addition, detailed studies using different times and/or dosages of CLA supplementation, at different ages, and in other metabolic tissues are necessary to confirm the ability of C9 and C10 to activate an inter-organ crosstalk in order to counteract the HFD-induced metabolic inflexibility.
